# Simple Mode of Checking Epistaxis

**Published:** 1877-08

**Authors:** 


Simple Mode of Checking Epistaxis.—The *Tribune Medicale* says that, even after plugging the nares, injection of perchloride of iron, etc., have failed, an emetic, given to the extent of producing vomiting, will permanently check epistaxis.

